# ​Occult extracranial malignancy after complete remission of pineal mixed germ cell tumors: a rare case report and literature review

**DOI:** 10.1186/s12887-023-04213-9

**Published:** 2023-09-07

**Authors:** Jun Liu, Luxiong Fang, Songtao Qi, Ye Song, Lei Han

**Affiliations:** 1 Department of Neurosurgery, Ganzhou People’s Hospital, Ganzhou, Jiangxi 341000 China; 2 Department of Neurosurgery, Institute of Brain Disease, Nanfang Hospital of Southern Medical University, Guangzhou, Guangdong 510515 China

**Keywords:** Intracranial germ cell tumor, Extracranial malignancy, Occult tumor, Tumor markers, Outcomes, Case report

## Abstract

**Background:**

​Extracranial metastasis can occur in intracranial germ cell tumors (GCTs), but it is very rare. Recurrence or metastasis of non-germinomatous germ cell tumors (NGGCTs) is often accompanied by elevated tumor markers. ​Occult extracranial metastases or recurrences with negative markers are often difficult to detect in time, resulting in a very poor prognosis.

**Case presentation:**

A 12-year-old boy was admitted to our institution with dizziness, headache, vomiting, and sleepiness. Magnetic resonance imaging (MRI) showed a pineal mass, accompanied by a significant increase in serum alpha-fetoprotein (AFP). The patient subsequently underwent total removal of the tumor. Pathology revealed that the tumor was a mixed GCT, consisting of mature teratoma, germinoma, and yolk sac tumor. Intracranial GCT achieved complete remission after intensive adjuvant chemotherapy and radiotherapy. Regular follow-up MRI revealed no recurrence of the intracranial tumor and continued monitoring of tumor markers revealed no abnormalities. ​Eight months later, the patient was readmitted due to progressive abdominal pain. Imaging and physical examination revealed abdominal occupation and lymphatic mass in the neck. He received salvage chemotherapy, anti-PD-1 immunotherapy, and palliative chemotherapy, but still developed multiple organ dysfunction syndromes (MODS) due to tumor progression and eventually died after one month.

**Conclusions:**

​This profound case suggests that intracranial NGGCTs may develop occult extracranial malignancy, which can be very severe at the time of clinical symptoms and has an extremely poor prognosis. Therefore, in addition to tumor marker monitoring, regular follow-up with extracranial imaging may be warranted to detect extracranial tumors as early as possible, although perhaps not as frequently as with neuroimaging.

## Introduction

Primary intracranial germ cell tumors (GCTs) may disseminate to the ventricular system or subarachnoid space of the spinal cord [1], however, their extra-neural metastasis is extremely rare. The extraneural metastasis may occur via ventriculoperitoneal shunt (VPS), hematogenous metastasis, or local invasion [2–4]. Among them, choriocarcinoma is the most common subtype of extra-neural metastasis [5]. For germinomas, bone metastasis is the most common site of extra-neural metastasis, followed by lung, lymph node, and soft tissue [6]. The most common extra-cranial locations of non-germinomatous germ cell tumors (NGGCTs) are the lung and mediastinum [5]. ​In addition, a small number of patients may develop metastases to the peritoneum, kidney, liver, scalp/cervical spine, bone and pancreas, spleen, bladder, and adrenal glands. Although intracranial GCTs may metastasize to the lymph nodes, the reported cases mainly involve patients with systemic metastasis.

​ Here, we described an intractable case who developed occult malignancies in the abdominal and cervical lymph nodes after comprehensive treatment of pineal mixed GCT with no recurrence at the primary site.

## Case presentation

**Fig. 1:**
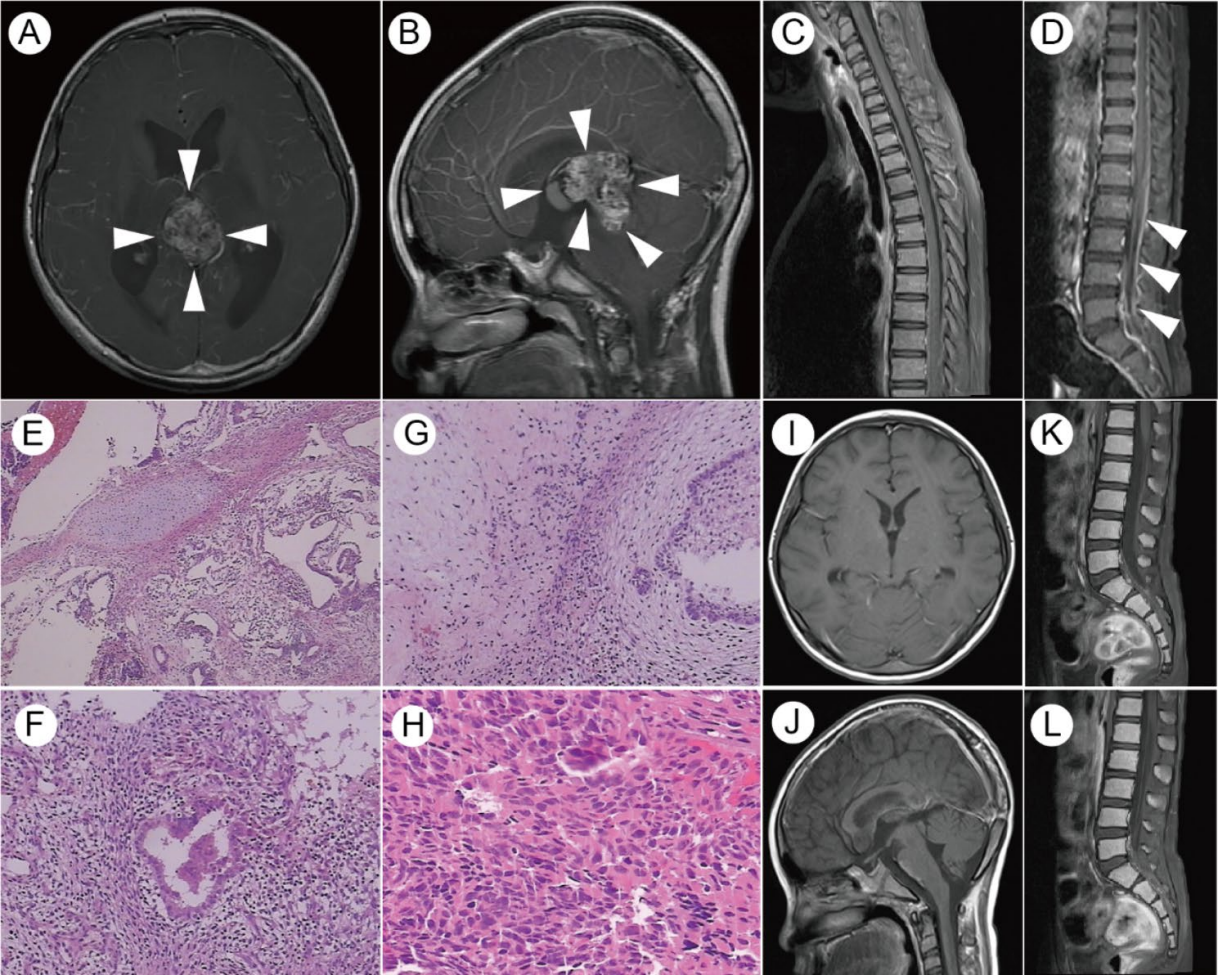
Imaging and histological features of central nervous system tumors in patients. **A**.​ T1 contrast-enhanced MRI reveals a heterogeneous pineal mass with obstructive hydrocephalus. **B**. Contrast-enhanced MRI (sagittal plane). **C-D**.​Contrast-enhanced MRI of the spine showing thickening and enhancement of the spinal dura in the cervical and thoracic segments. **E-H**. Hematoxylin and eosin (H&E) staining of tumor tissue in the pineal region. (E. Original magnification **100×**; F. Original magnification **200×**; G. Original magnification **200×**; H. Original magnification **400×**.) **I-J**. No residual or recurrent tumor was observed in the pineal region after treatment. **K-L**. The thickening and enhancement of the dura mater in the original lumbosacral spinal canal were eliminated after chemotherapy

A 12-year-old boy presented to our hospital with a 10-day history of dizziness, headache, vomiting, and sleepiness. He had no personal medical history or family history of genetic diseases or tumors. Magnetic resonance imaging (MRI) showed uneven enhancement of space-occupying lesions in the pineal region (Fig. 1A, B). The spinal MRI revealed thickening and enhancement of the spinal dura (Fig. 1 C, D). The serum alpha-fetoprotein (AFP) and beta-subunit human chorionic gonadotropin (β-HCG) values were 872.5 ug/L and 0.47 IU/L, respectively. ​The patient was clinically diagnosed with pineal mixed GCT, which was subsequently resected via the occipital tentorial approach. Histological examination (Fig. 1E-H) revealed that the mixed GCT was composed of 50% mature teratoma, 30% germinoma, and 20% yolk sac tumor components. On the tenth day after surgery, chemotherapy was started for a total of six courses (three courses of cisplatin 20 mg/m^2^ plus etoposide 100 mg/m^2^ for 1–5 days, and three courses of ifosfamide 1500 mg/m^2^ plus etoposide 100 mg/m^2^ for 1–5 days). Subsequently, the patient also received cerebrospinal radiotherapy (CSI) (23.4 Gy) and increased local irradiation with PCTV1 (57.4 Gy) and PCTV2 (54 Gy). A normal range of the serum AFP (0.4–2.3 ug/L) and β-HCG (less than 0.10 IU/L) was observed from six weeks to seven months after the operation. A re-examination of the MRI showed that there was no residual tumor in the pineal region (Fig. 1I, J), and the thickening and enhancement of the lumbosacral dura were eliminated (Fig. 1K-L). The intracranial GCTs achieved complete remission.

**Fig. 2:**
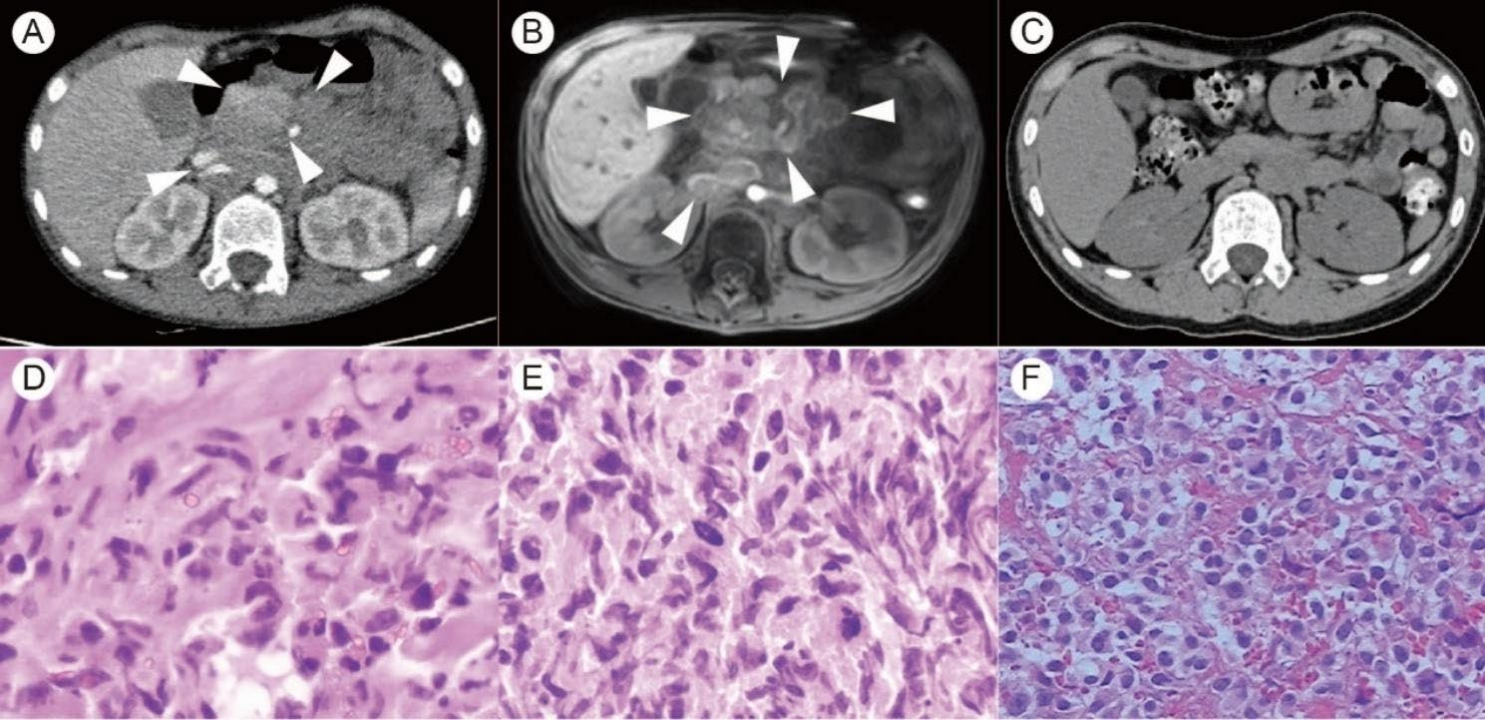
Imaging and histological features of metastatic extra-neural tumors. **A**. Contrast-enhanced CT scan showing multiple soft tissue shadows in the left upper abdomen and retroperitoneum. **B**. Contrast-enhanced MRI showing mild annular enhancement. **C**. The whole abdominal computerized tomography (CT) scan before pineal GCT treatment. **D-E.** H&E staining of mesenteric lymph node tissue from a biopsy (**400×**). **F**. H&E staining of cervical lymph node tissue (**400×**)

However, the patient was readmitted because of progressive abdominal pain and distention eight months after surgery. Computerized tomography (CT) showed multiple soft tissue shadows in the left upper abdomen and retro-peritoneum (Fig. 2A). The lesions were detected surrounding the abdominal trunk, upper mesenteric arteries and veins, and both the renal arteries and veins. The pancreas was pushed forward and had no clear boundaries with the lesions. MRI showed partial fusion of the lesion. Contrast->enhanced MRI revealed mild annular enhancement (Fig. 2B). However, a whole abdominal CT scan before the treatment of pineal GCT had shown no obvious abnormalities (Fig. 2 C). Initial testicular ultrasound and whole abdominal CT showed no testicular or groin abnormalities. The serum AFP and β-HCG values were detected within their normal range at 2.10 ug/L and less than 0.10 IU/L, respectively. The compression of the mass caused ileus and the patient was unable to eat. He then underwent laparotomy, double-barrel transverse colostomy, abdominal lymph node biopsy, and intestinal adhesion release. Histological (Fig. 2D, E) and immunohistochemical (IHC) (Table 1) staining of biopsied lymph nodes revealed a malignant tumor, which was considered a lymphatic metastasis of malignant mixed GCTs. However, the typical cell morphology of the GCTs component was not seen in this tumor tissue. The tumor was progressing rapidly and a cervical lymph node biopsy was performed to confirm the diagnosis. Histological revealed the presence of a necrotic malignant tumor (Fig. 2F). Tumors of lymphoid hematopoietic origin were excluded combined with the IHC findings (Table 1). The metastasis of mesenchymal or epithelial malignancies other than germinoma in mixed GCTs was considered. The patient received one course of salvage chemotherapy with cisplatin and etoposide, which had to be terminated due to the patient’s intolerance to the treatment which led to labored breathing and decreased blood oxygen saturation. The patient could no longer tolerate salvage chemotherapy due to poor general condition. The patient’s family strongly insisted on palliative chemotherapy, despite having been advised that it was of little value. Anti-PD-1 therapy was administered in view of reported cases of response to anti-PD-1 antibodies in patients with platinum-resistant recurrent GCTs. Therefore, toripalimab at a dose of 100 mg, palliative chemotherapy with temozolomide (TMZ), and capecitabine were also administered. Unfortunately, the outcomes of the treatment weren’t successful, and the patient subsequently developed hypovolemic shock and multiple organ dysfunction syndromes (MODS) due to exophytic growth of the abdominal tumor, resulting in elevated abdominal pressure. Further treatment was waived, and discharge was requested. One day after discharge, the child’s condition deteriorated, and he died.

**Table 1:** Summary of immunohistochemical staining

Variable	Tissue specimens		
	Pineal tumor	Mesenteric lymph nodes	Cervical lymph node
PLAP	Partial +	-	-
OCT-4	Partial +	-	-
CD117	Partial +	Partial +	-
AFP	+	-	±
HCG	-	-	ND
SALL4	Partial +	-	-
CK	+ / Partial + ^a^	Partial +/ -^b^	+
Vim	+	ND	+
Ki-67	+, 40% / 60%^a^	+, 80% / 30%^b^	+, 85%
GFAP	Partial +	-	ND
Oligo 2	ND	-	ND
PHOX IIb	ND	-	ND
Syn	-	Local +	ND
CgA	-	-	ND
Actin	-	ND	ND
Desmin	-	ND	-
MyoD1	-	-	ND
Myogenin	-	ND	ND
EMA	ND	ND	-
Mutant p53	ND	Diffuse strong +	ND
S-100	ND	Diffuse +	+
INF1	ND	No loss	ND
SOX-10	ND	-	-
CD34	ND	-	-
SMA	ND	ND	-
LCA	ND	Partial +	ND
TTF	ND	-	ND
CD1a	ND	ND	-
CD3	ND	Sporadic + (lymphocyte)	-
CD5	ND	ND	-
CD20	ND	-	-
CD30	-	Sporadic +	Local ±
CD35	ND	ND	-
CD56	Partial +	+	ND
CD57	ND	Partial +	ND
CD68	ND	ND	Sporadic +
CD79α	ND	ND	-
CD99	ND	-	-
Bcl-2	ND	ND	-
ALK	ND	-	-
GPC-3	ND	-	ND
Hepatocyte	ND	-	ND
EBER (FISH)	ND	ND	-

a: Represents proteins with different staining results between two tissue specimens. b: The latter data are IHC staining results from other hospitals. +: Positive; -: Negative; ±: Weakly positive; ND: Not done

## Discussion

Intracranial GCTs can be disseminated to the ventricle or spinal cord but rarely exhibits extra-neural metastasis [1, 6]. ​Metastases occur mainly through the VPS and blood, with incidence rates of approximately 10% and 3–5%, respectively [2–4]. Extracranial metachronous GCTs have also been reported at long intervals after treatment of intracranial GCTs [7, 8]. Most cases of extracranial metastases are NGGCTs, of which choriocarcinoma is the most frequent one [5]. In a review, Watterson et al. [5] showed that the lungs and mediastinum were the most common sites of metastasis (27 cases), whereas lymph node metastasis was found in only three cases. In addition, all six patients with peritoneal metastases underwent VPS in their study. Overall, the specific route of extraneural metastasis is still unclear, and it may occur through multiple routes, mainly including the blood route, lymphatic route, cerebrospinal fluid implantation, and seeding caused by the surgical invasion. In addition, prolonged chemo-radiotherapy that reduces the density of the meninges may also cause extracranial metastasis.

In this case, the patient underwent total removal of pineal GCTs followed by systematic chemotherapy and irradiation. Subsequently, abdominal and cervical lymph node tumors were discovered one month after the end of treatment due to progressive abdominal pain. The tumors were first considered as metastasis due to a history of intracranial GCTs. Invasive surgery may allow the infiltration of tumor cells into the scalp, allowing them to enter the blood or lymphatic system. Craniotomy and shunts may lead to distant metastasis through blood vessels by disrupting the blood-brain barrier. In addition, tumor growth into cranial structures can present with lymphatic system implantation. Since the patient was not treated with VPS; therefore, it was unlikely to have tumor metastasized through VPS. The absence of a tumor in the lungs suggested the unlikelihood of hematogenous metastasis to the abdominal cavity and cervical lymph nodes. The absence of a tumor in the skull makes lymphatic metastasis through invasion of the skull unlikely. Although the patient was suspected to have spinal cord dissemination before treatment, the abnormal enhancement of the spinal cord exhibited complete remission after chemotherapy. MRI showed no recurrence or fusion of the abdominal lesions, therefore metastasis was unlikely to have occurred through local invasion. Histopathology of both abdominal and cervical lymph node biopsies showed the absence of typical GCTs components. As an abdominal mass, an abdominal primary tumor should be also considered.​ Therefore, there can be several potential origins of the tumor: (1) GCT metastasized via the direct lymphatic system but no GCT components were obtained by biopsy; (2) tumor was a mesenchymal or epithelial malignant tumor metastasized in the intracranial GCT components; and/or (3) it was a new tumor.

Histological morphology of the cervical lymph node biopsy revealed malignancy with necrosis. ​The patient had pleural and abdominal effusions and rapid tumor progression, which indicated malignant manifestations. These features suggest the possibility of malignant tumors in the abdominal cavity and cervical lymph nodes. Hematopoietic tumors were excluded from IHC analyses. IHC findings of the abdominal lymph nodes showed that the mutant p53 protein was diffusely expressed. Although p53 is overexpressed in approximately 94% of intracranial GCTs [9] and wild-type p53 is also found in testicular GCTs, [10] p53 mutations have not been previously reported. However, p53 mutations could also have occurred in the primary mediastinal GCTs. Akizuki et al. [11] identified *TP53* mutations and acute myeloid leukemia in all three patients with mediastinal GCTs using whole-exome sequencing. Thus, suggesting that patients with *TP53* mutations are more likely to develop multiple tumors. Li-Fraumeni syndrome (LFS) is a cancer predisposition syndrome, wherein ​regardless of the sequence of tumor occurrence, multiple cancers can occur over a short period [12, 13]. The germline *TP53* variant is the only identified gene associated with LFS [12]. LFS-associated brain tumors can occur in childhood and adulthood, with a median age of 16 years old [14]. ​Therefore, LFS should also be identified after the presence of an ectopic secondary tumor.

In this case, IHC staining of biopsied tissues from the cervical lymph node demonstrated weakly positive AFP protein. IHC results of the pineal and extracranial tumors of lymph nodes showed positive CK and CD117 expression, suggesting that the tumors had the characteristics of bidirectional differentiation. In addition, both abdominal tumors and pineal tumors contained mesenchymal components. Therefore, abdominal tumors may be derived from mesenchymal and sarcomatous components of pineal lesions. In this patient, metastasis of the mesenchymal component of the pineal tumor through the lymphatic route was considered, but the mechanism of metastasis was unclear.

Intracranial GCTs with extracranial metastases usually have a poor prognosis. Watterson et al. [5] reported 32 fatal cases of intracranial NGGCT with extracranial metastasis. They reported a case of mixed GCT extracranial metastases that had progression-free survival of 46 months after treatment. This was the longest survival among the 33 patients with GCT extracranial metastasis. In the present case, the patient developed MODS due to tumor progression at one month of disease onset despite salvage therapy. The disease progressed so rapidly that there was no more time for (and could not be tolerated) additional salvage therapies. Metastatic recurrence of NGGCTs is often accompanied by the elevation of tumor markers AFP and/or β-HCG [15], but in this case, continuous monitoring of serum markers after treatment was always negative. Considering the possibility of multiple intracranial and extracranial GCTs at the same time, when considering intracranial GCTs in our institution, a whole abdominal CT is usually performed. ​However, if the initial examination is normal, a regular follow-up abdominal CT or MRI is not usually performed after treatment. As a result, the recurrence of extracranial malignancy in this patient was very occult. The profound lessons of this case suggest that regular follow-up abdominal imaging may be needed for intracranial GCTs, and especially for NGGCTs, although not as frequently as neuroimaging. Regular follow-up with abdominal imaging is conducive to the early detection of metastatic lesions and more time for treatment. At the same time, patients are better able to tolerate salvage therapy, which is more likely to improve survival outcomes.

In conclusion, we report a rare case of a mixed GCT in the pineal region that developed extracranial metastasis after treatment. It is characterized by complex diagnosis, metastasis of atypical GCTs components, insensitive chemo- and immunotherapy, rapid tumor progression, and poor prognosis. Extracranial metastasis of intracranial GCTs is very rare. The clinical manifestations and imaging lack specificity. Especially in the case of negative tumor markers, the diagnosis is difficult, and the final diagnosis depends on histopathological examination. This case suggests the possibility of extraneural metastasis of intracranial GCT, which may be the atypical GCTs component. Further research is required to elucidate the pathogenesis of such complex tumors and develop novel therapeutics to improve the prognosis in such rare cases. The profound lessons of this case suggest that regular follow-up abdominal imaging may be needed for NGGCTs, although not as frequently as neuroimaging.

## Data Availability

Data sharing is not applicable to this article, as no datasets were generated or analyzed during the current study.

## Abbreviations

GCTs: Germ cell tumors
NGGCTs: Non-germinomatous germ cell tumors
MRI: Magnetic resonance imaging
AFP: Alpha-fetoprotein
MODS: Multiple organ dysfunction syndromes
VPS: Ventriculoperitoneal shunt
β-HCG: Beta-subunit human chorionic gonadotropin
CT: Computerized tomography
IHC: Immunohistochemical
TMZ: Temozolomide
LFS: Li-Fraumeni syndrome
